# Mesenchymal stromal cells suppress synovial fluid-derived t cells from juvenile idiopathic arthritis patients *in vitro*

**DOI:** 10.1186/1546-0096-12-S1-P129

**Published:** 2014-09-17

**Authors:** J Wienke, S de Roock, J Swart, A Martens, N Wulffraat, B Prakken

**Affiliations:** 1 Laboratory of Translational Immunology, UMC Utrecht, Utrecht, Netherlands

## Introduction

Mesenchymal stromal cells (MSC) are multipotent cells with an immunosuppressive capacity. In the last decade the feasibility and safety of MSC therapy has been established in various inflammatory diseases, including graft versus host disease. MSC are known to suppress T cell function and modulate T helper 17 (Th17) cells and regulatory T cells (Tregs), which play an important role in the pathogenesis of juvenile idiopathic arthritis (JIA). MSC may therefore serve as a new treatment option for JIA patients refractory to conventional therapies. However, it is unknown whether MSC are capable of suppressing the highly inflammatory synovial fluid mononuclear cells (SFMC), which have been shown to be resistant to suppression by Tregs. Furthermore, the effect of the inflammatory environment on MSC function is largely unknown, though it has been suggested that proinflammatory cytokines can enhance the suppressive potential of MSC.

## Objectives

We aimed to study the *in vitro* immunomodulatory effects of MSC on SFMC from JIA patients, with a focus on T cell function. In addition, we assessed the influence of the inflammatory micro-environment, i.e. monocytes and proinflammatory cytokines, on the suppressive potential of MSC.

## Methods

MSC were cocultured with either peripheral blood mononuclear cells (PBMC) or synovial fluid mononuclear cells (SFMC) from JIA patients (paired samples) or PBMC from healthy controls. We analyzed the effect of MSC on T cell proliferation and Th17 and Treg numbers by flow cytometry. Cytokine production was analyzed in the culture supernatant by Luminex assay. Furthermore, we depleted monocytes from culture and blocked the proinflammatory cytokines TNFα, IFNγ, IL-1β and IL-6 to assess the result on MSC-mediated suppression.

## Results

MSC suppressed proliferation of PB T cells and SF T cells from JIA patients dose-dependently, but patient-derived T cells were less susceptible to suppression than healthy donor-derived T cells. MSC reduced Th17 numbers and increased Treg numbers in PBMC, but not in SFMC. Addition of MSC did not clearly affect IL-17 levels in the supernatant, but TNFα production was suppressed in both PBMC and SFMC. Contrary to previous reports, blockade of TNFα, IFNγ, IL-1β and IL-6 during *in vitro* culture did not reduce suppression, but rather enhanced suppression in PBMC from JIA patients. Similarly, depletion of monocytes increased MSC-dependent suppression of healthy control PB T cells.

## Conclusion

MSC suppress proliferation of synovial fluid T cells from JIA patients in a dose-dependent manner. However, patient-derived T cells are less susceptible to immunomodulation than healthy donor T cells. We propose that reduction of proinflammatory stimuli in conjunction with MSC therapy may benefit the suppressive effects of MSC in JIA.

## Disclosure of interest

None declared.

